# Supplementary material for: The neurotranscriptome of the Aedes aegypti mosquito
Source: BMC Genomics. 2016 Jan 6;17:32. doi: 10.1186/s12864-015-2239-0 (PMC4704297; doi:10.1186/s12864-015-2239-0)

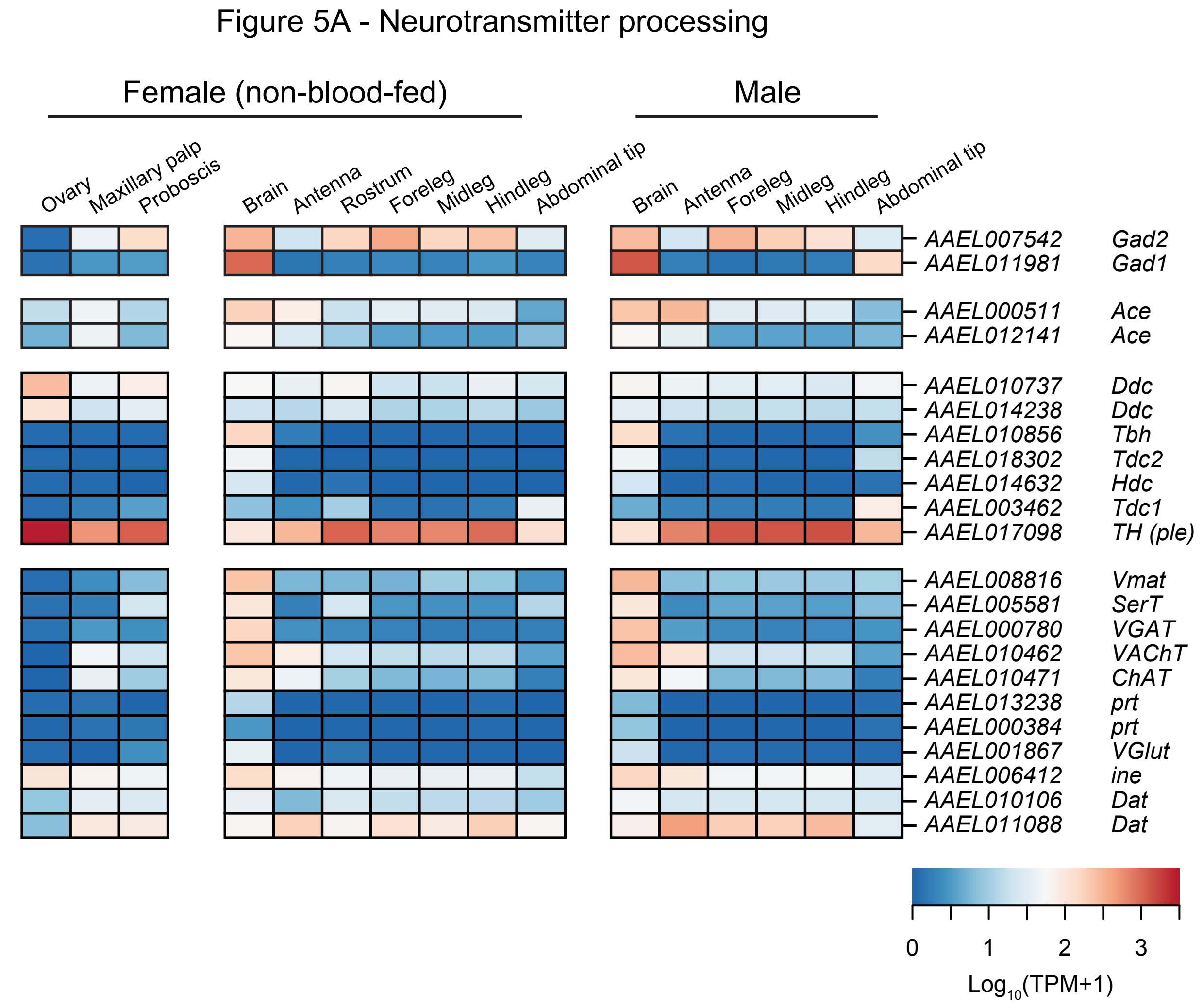
Figure 5A - Neurotransmitter processing
Female (non-blood-fed)
Male
Ovary
Maxillary palp
Proboscis
Brain
Antenna
Rostrum
Foreleg
Midleg
Hindleg
Abdominal tip
Brain
Antenna
Foreleg
Midleg
Hindleg
Abdominal tip
AAEL007542 Gad2
AAEL011981 Gad1
AAEL000511 Ace
AAEL012141 Ace
AAEL010737 Ddc
AAEL014238 Ddc
AAEL010856 Tbh
AAEL018302 Tdc2
AAEL014632 Hdc
AAEL003462 Tdc1
AAEL017098 TH (ple)
AAEL008816 Vmat
AAEL005581 SerT
AAEL000780 VGAT
AAEL010462 VAChT
AAEL010471 ChAT
AAEL013238 prt
AAEL000384 prt
AAEL001867 VGlut
AAEL006412 ine
AAEL010106 Dat
AAEL011088 Dat
0
1
2
3
Log10(TPM+1)

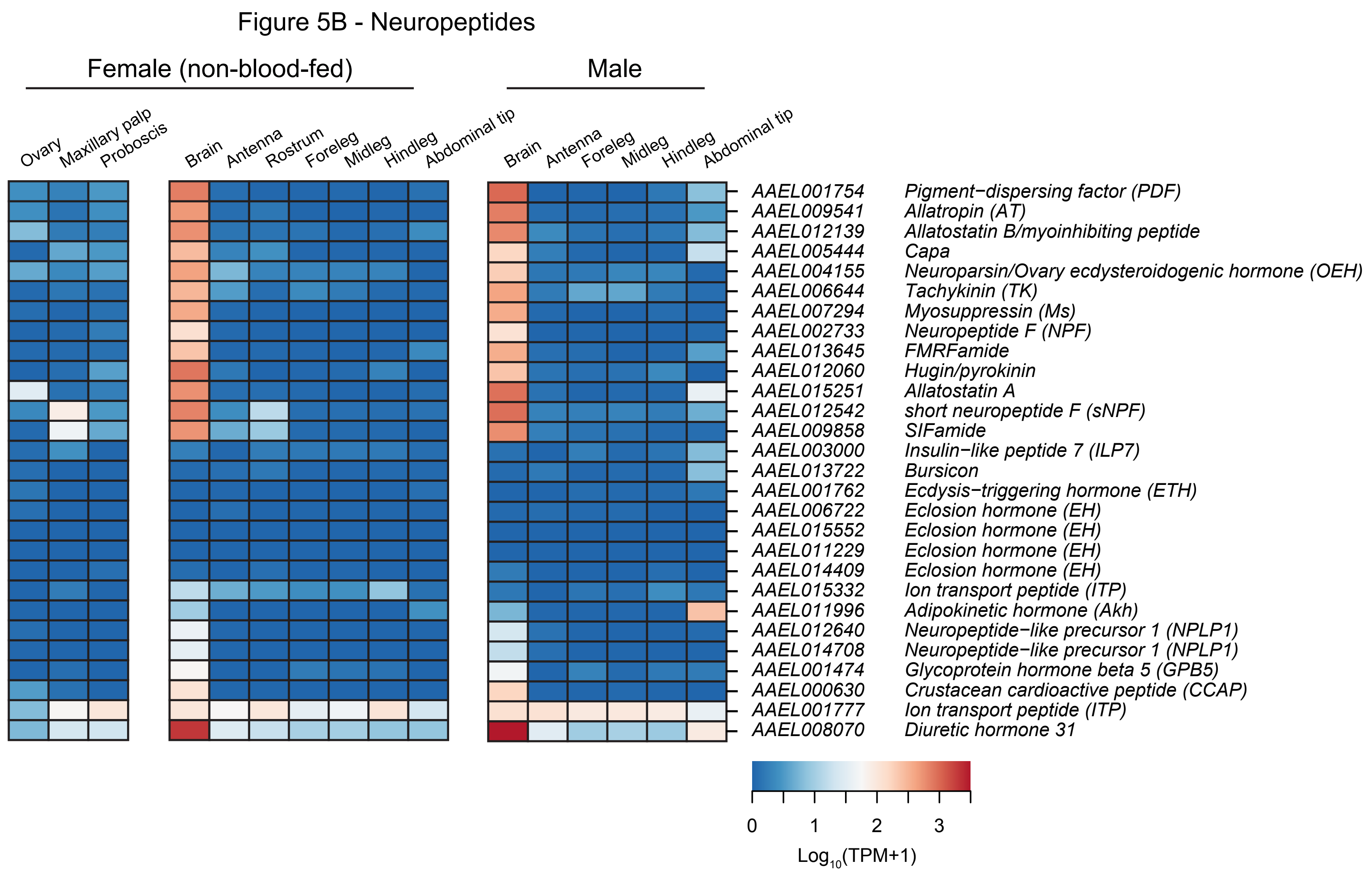
Figure 5B - Neuropeptides
Female (non-blood-fed)
Male
Ovary
Maxillary palp
Proboscis
Brain
Antenna
Rostrum
Foreleg
Midleg
Hindleg
Abdominal tip
Brain
Antenna
Foreleg
Midleg
Hindleg
Abdominal tip
AAEL001754 Pigment−dispersing factor (PDF)
AAEL009541 Allatropin (AT)
AAEL012139 Allatostatin B/myoinhibiting peptide
AAEL005444 Capa
AAEL004155 Neuroparsin/Ovary ecdysteroidogenic hormone (OEH)
AAEL006644 Tachykinin (TK)
AAEL007294 Myosuppressin (Ms)
AAEL002733 Neuropeptide F (NPF)
AAEL013645 FMRFamide
AAEL012060 Hugin/pyrokinin
AAEL015251 Allatostatin A
AAEL012542 short neuropeptide F (sNPF)
AAEL009858 SIFamide
AAEL003000 Insulin−like peptide 7 (ILP7)
AAEL013722 Bursicon
AAEL001762 Ecdysis−triggering hormone (ETH)
AAEL006722 Eclosion hormone (EH)
AAEL015552 Eclosion hormone (EH)
AAEL011229 Eclosion hormone (EH)
AAEL014409 Eclosion hormone (EH)
AAEL015332 Ion transport peptide (ITP)
AAEL011996 Adipokinetic hormone (Akh)
AAEL012640 Neuropeptide−like precursor 1 (NPLP1)
AAEL014708 Neuropeptide−like precursor 1 (NPLP1)
AAEL001474 Glycoprotein hormone beta 5 (GPB5)
AAEL000630 Crustacean cardioactive peptide (CCAP)
AAEL001777 Ion transport peptide (ITP)
AAEL008070 Diuretic hormone 31
0
1
2
3
Log10(TPM+1)

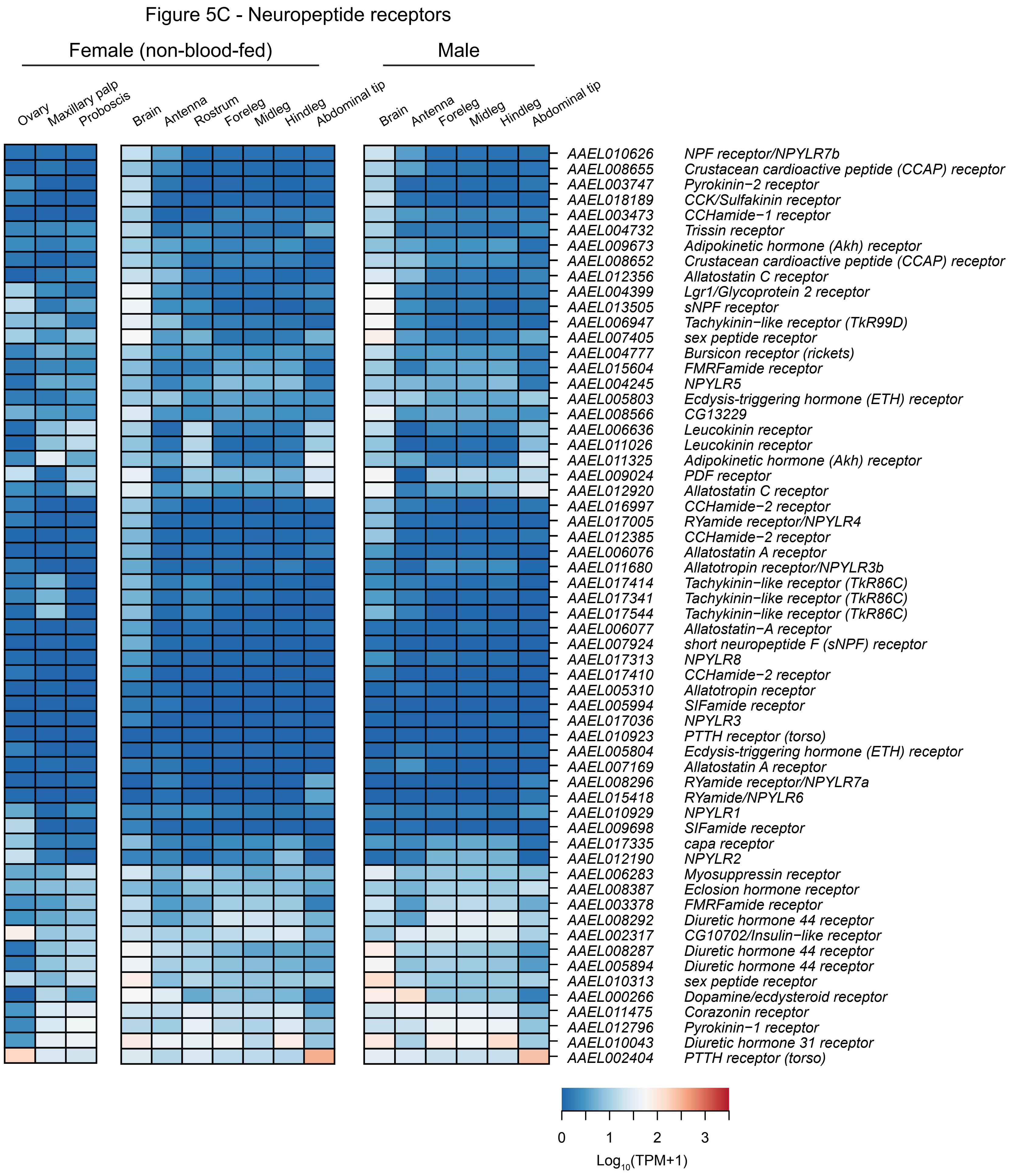
Figure 5C - Neuropeptide receptors
Female (non-blood-fed)
Male
Ovary
Maxillary palp
Proboscis
Brain
Antenna
Rostrum
Foreleg
Midleg
Hindleg
Abdominal tip
Brain
Antenna
Foreleg
Midleg
Hindleg
Abdominal tip
AAEL010626 NPF receptor/NPYLR7b
AAEL008655 Crustacean cardioactive peptide (CCAP) receptor
AAEL003747 Pyrokinin−2 receptor
AAEL018189 CCK/Sulfakinin receptor
AAEL003473 CCHamide−1 receptor
AAEL004732 Trissin receptor
AAEL009673 Adipokinetic hormone (Akh) receptor
AAEL008652 Crustacean cardioactive peptide (CCAP) receptor
AAEL012356 Allatostatin C receptor
AAEL004399 Lgr1/Glycoprotein 2 receptor
AAEL013505 sNPF receptor
AAEL006947 Tachykinin−like receptor (TkR99D)
AAEL007405 sex peptide receptor
AAEL004777 Bursicon receptor (rickets)
AAEL015604 FMRFamide receptor
AAEL004245 NPYLR5
AAEL005803 Ecdysis-triggering hormone (ETH) receptor
AAEL008566 CG13229
AAEL006636 Leucokinin receptor
AAEL011026 Leucokinin receptor
AAEL011325 Adipokinetic hormone (Akh) receptor
AAEL009024 PDF receptor
AAEL012920 Allatostatin C receptor
AAEL016997 CCHamide−2 receptor
AAEL017005 RYamide receptor/NPYLR4
AAEL012385 CCHamide−2 receptor
AAEL006076 Allatostatin A receptor
AAEL011680 Allatotropin receptor/NPYLR3b
AAEL017414 Tachykinin−like receptor (TkR86C)
AAEL017341 Tachykinin−like receptor (TkR86C)
AAEL017544 Tachykinin−like receptor (TkR86C)
AAEL006077 Allatostatin−A receptor
AAEL007924 short neuropeptide F (sNPF) receptor
AAEL017313 NPYLR8
AAEL017410 CCHamide−2 receptor
AAEL005310 Allatotropin receptor
AAEL005994 SIFamide receptor
AAEL017036 NPYLR3
AAEL010923 PTTH receptor (torso)
AAEL005804 Ecdysis-triggering hormone (ETH) receptor
AAEL007169 Allatostatin A receptor
AAEL008296 RYamide receptor/NPYLR7a
AAEL015418 RYamide/NPYLR6
AAEL010929 NPYLR1
AAEL009698 SIFamide receptor
AAEL017335 capa receptor
AAEL012190 NPYLR2
AAEL006283 Myosuppressin receptor
AAEL008387 Eclosion hormone receptor
AAEL003378 FMRFamide receptor
AAEL008292 Diuretic hormone 44 receptor
AAEL002317 CG10702/Insulin-like receptor
AAEL008287 Diuretic hormone 44 receptor
AAEL005894 Diuretic hormone 44 receptor
AAEL010313 sex peptide receptor
AAEL000266 Dopamine/ecdysteroid receptor
AAEL011475 Corazonin receptor
AAEL012796 Pyrokinin−1 receptor
AAEL010043 Diuretic hormone 31 receptor
AAEL002404 PTTH receptor (torso)
0
1
2
3
Log10(TPM+1)

Figure 6A - Odorant receptors (ORs)

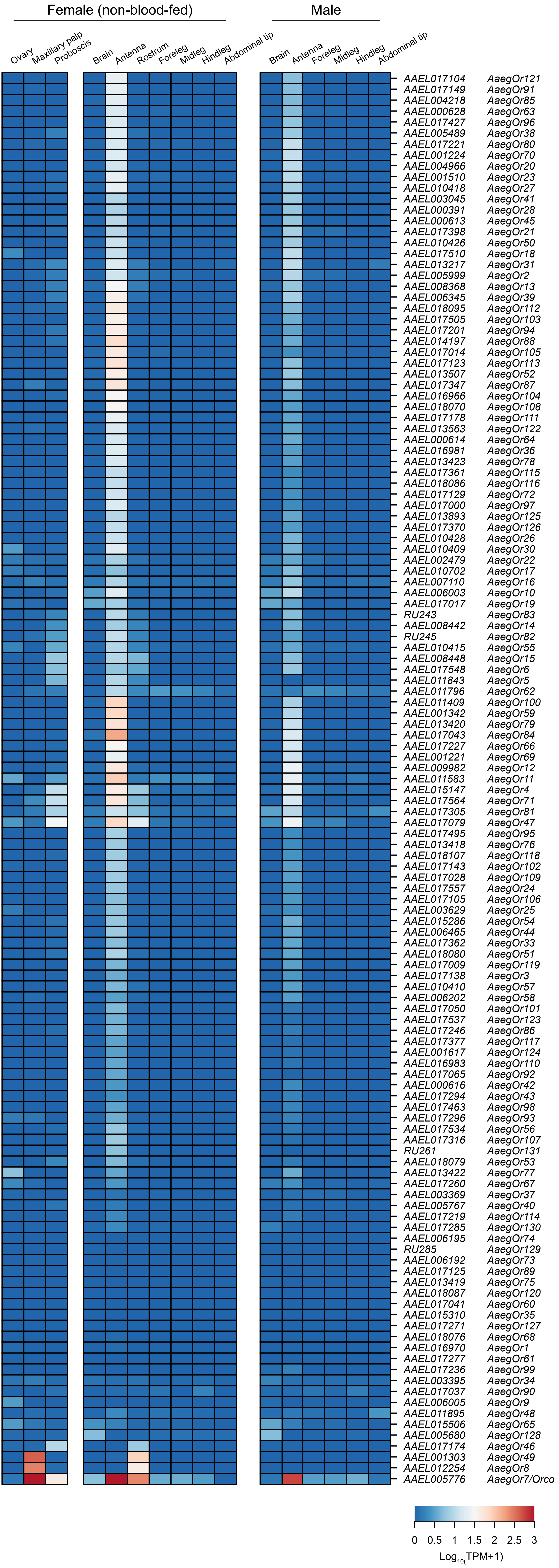

Figure 6B- Odorant binding proteins (OBPs)

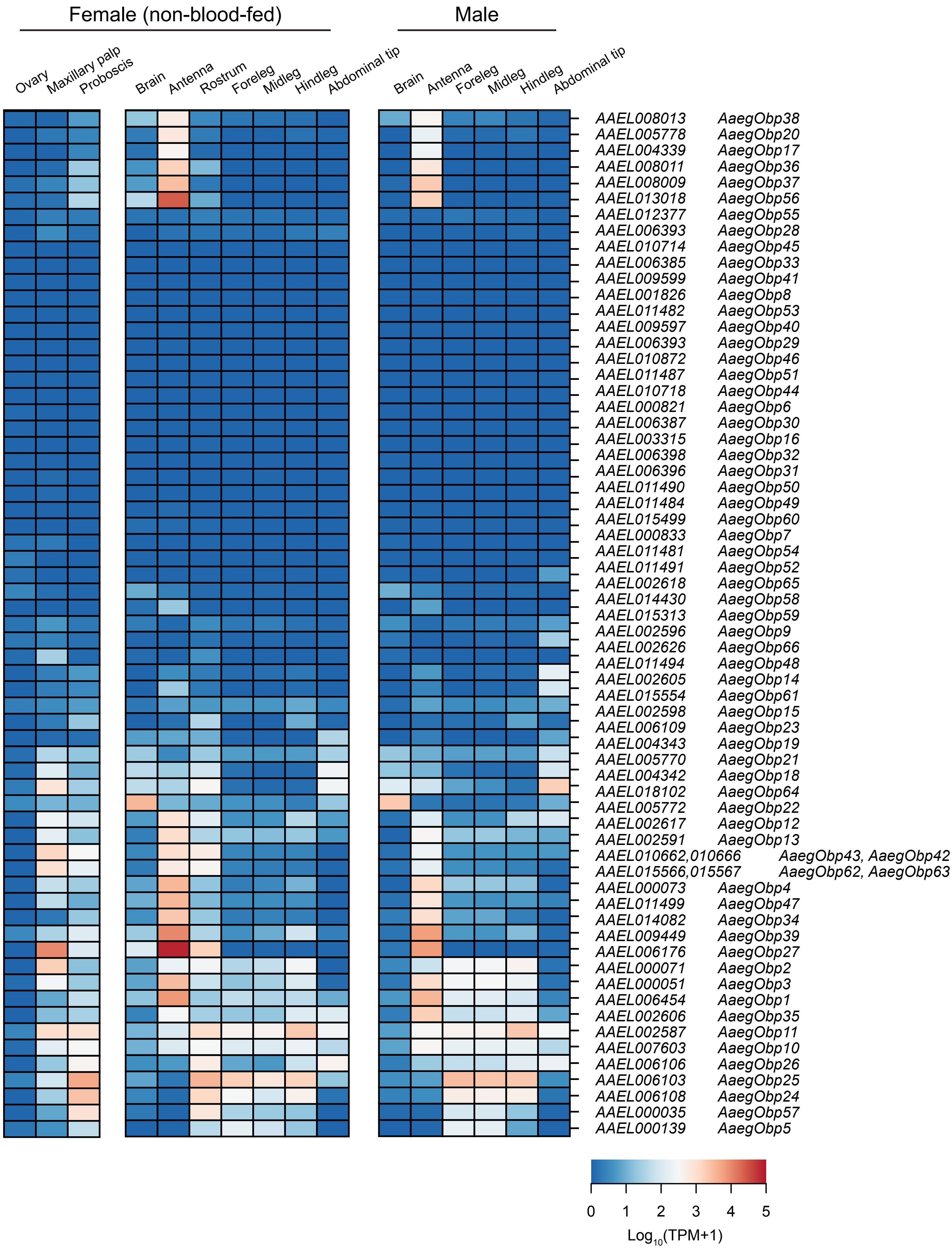

Figure 7A - Ionotropic receptors (IRs)

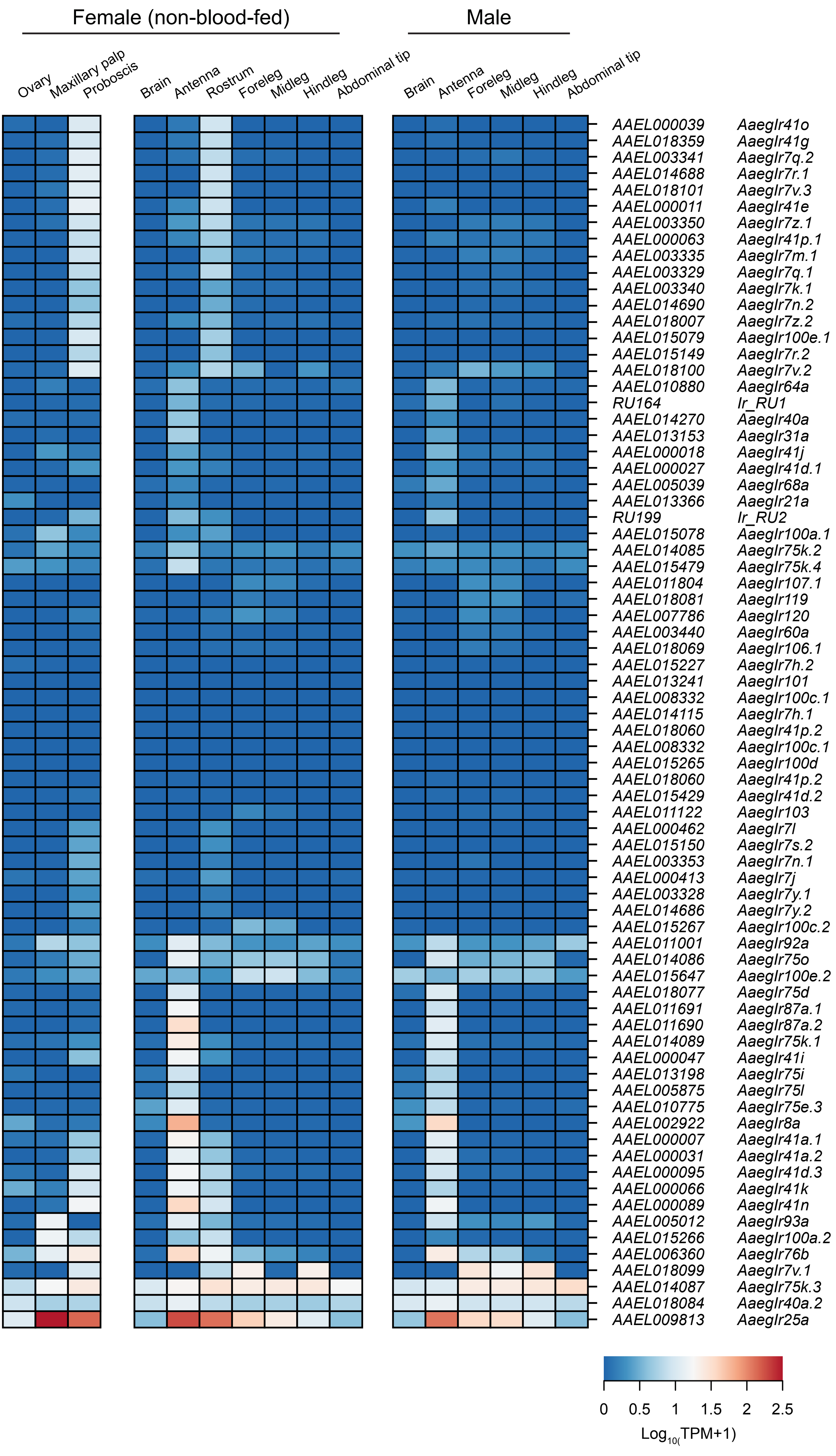

Figure 7B - Gustatory receptors (GRs)

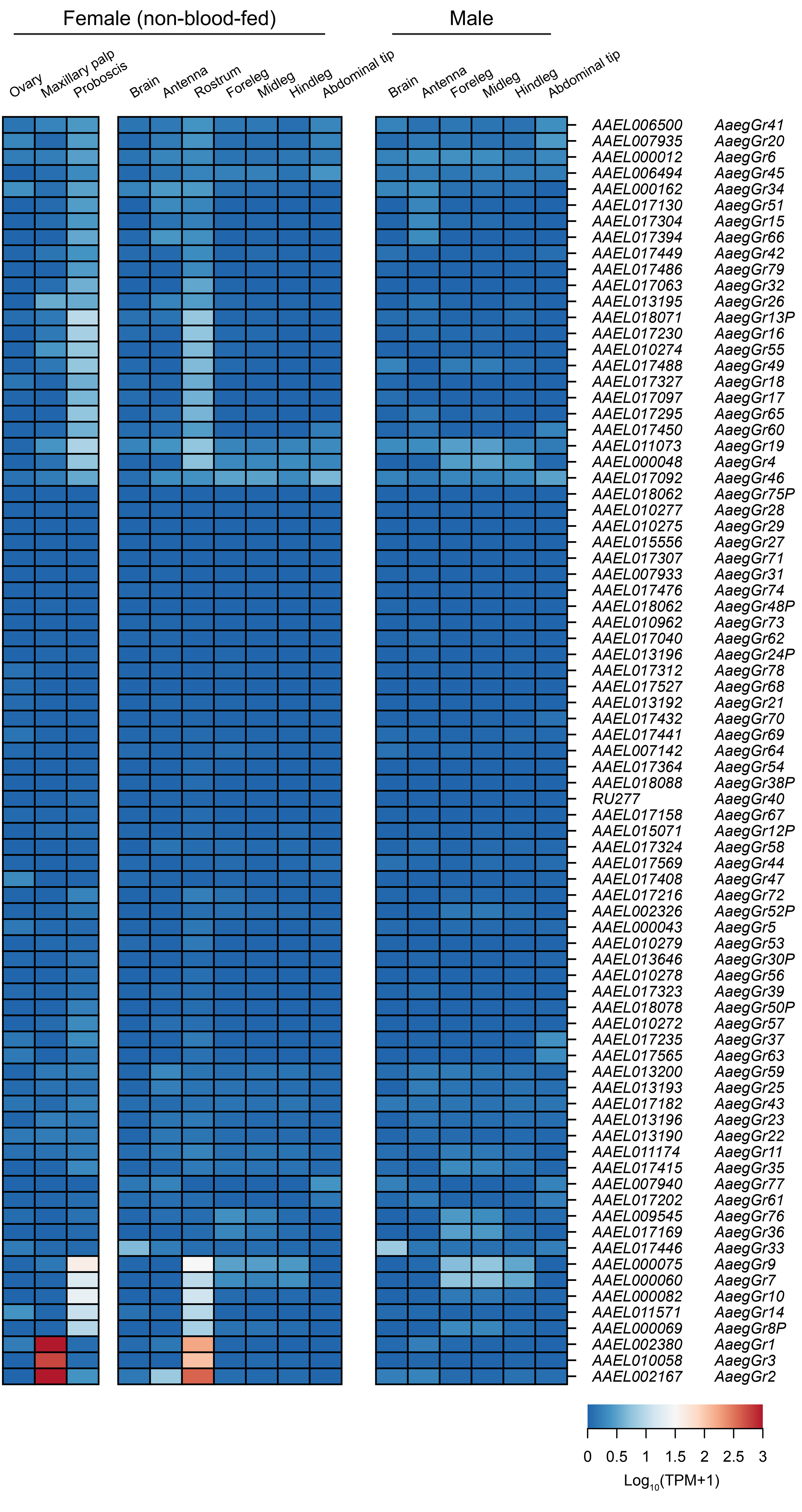

Figure 7C - pickpocket (PPK) channels

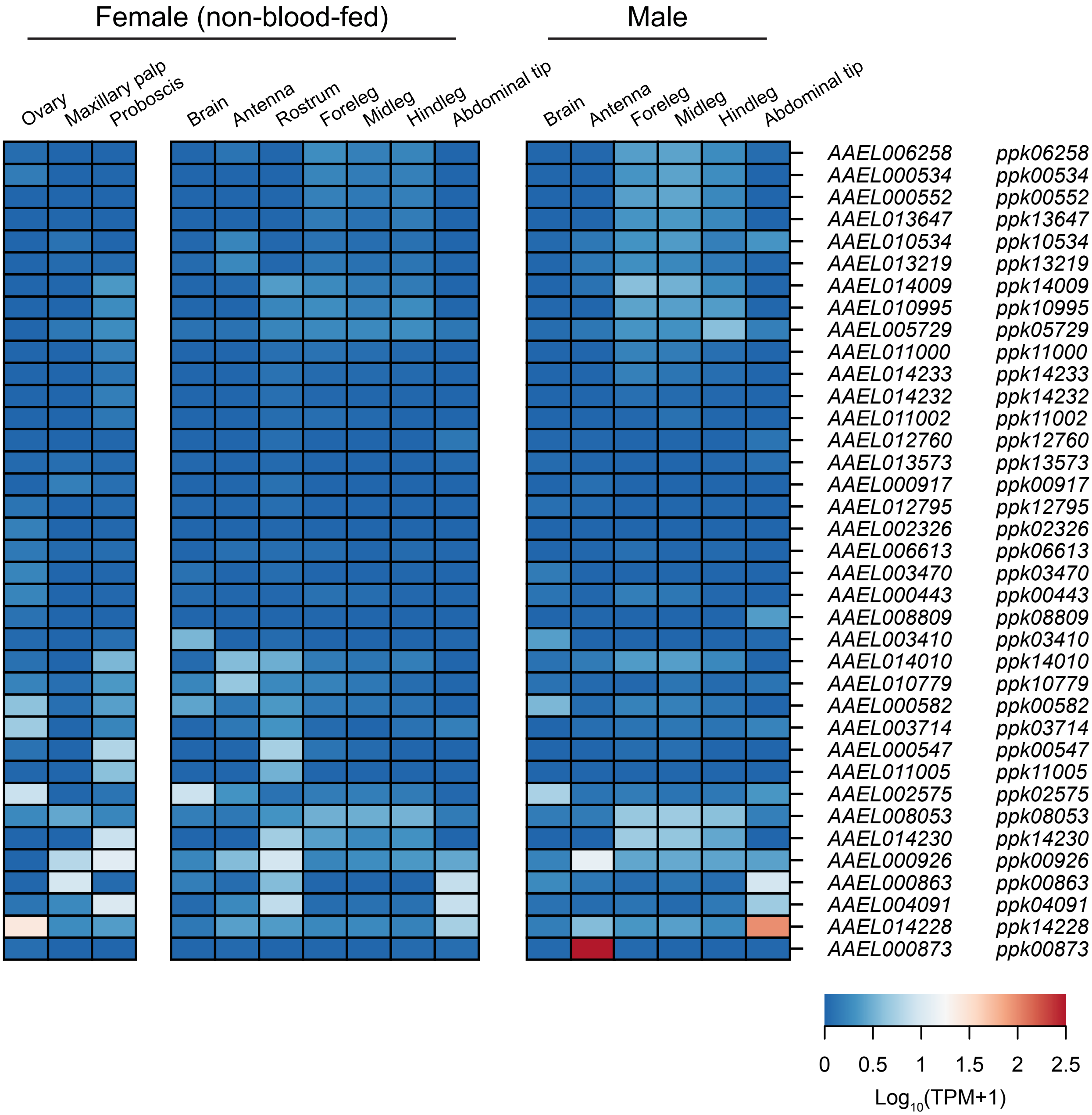

Figure 7D - TRP channels

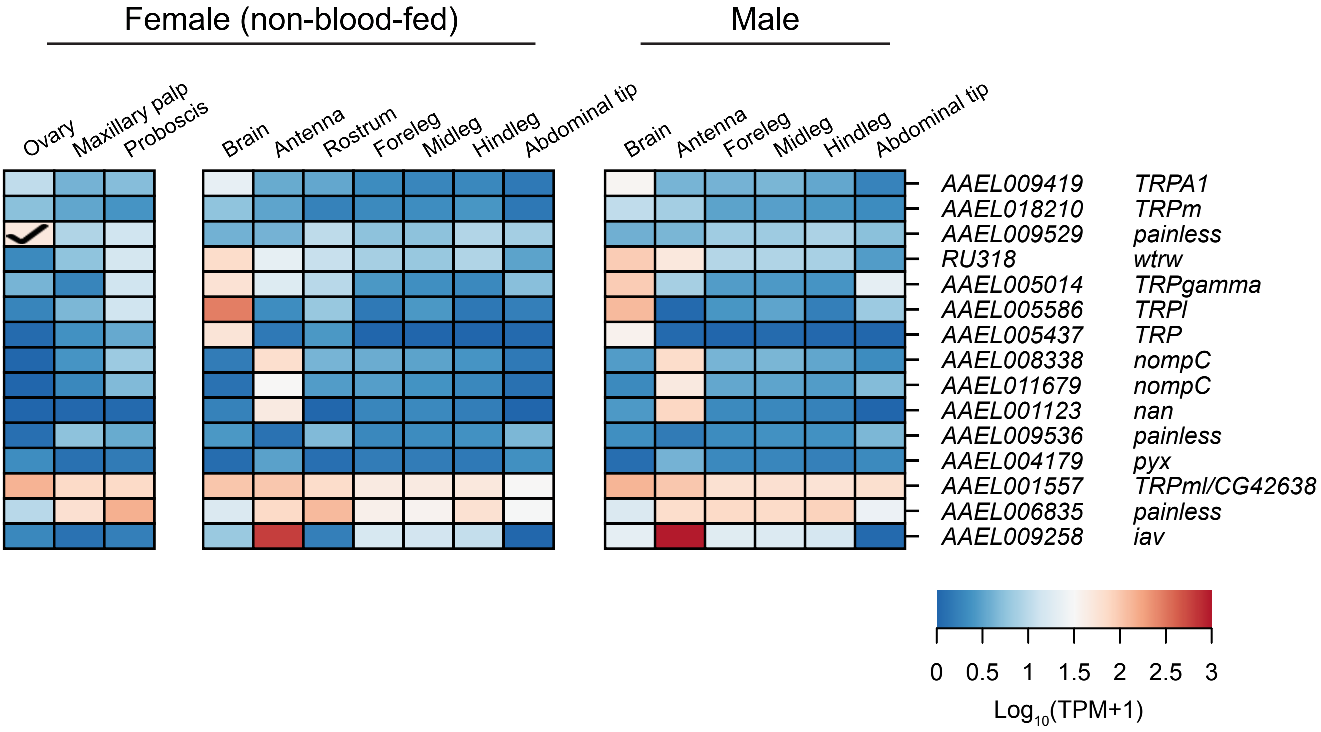

Supplement: Additional file 7: — Shows an expansion of expression heat-maps from Figs. 5 to 7 showing gene names for individual rows. (PDF 463 kb) [file 12864_2015_2239_MOESM7_ESM.pdf]
