# Supplementary figures and images for: The neurotranscriptome of the Aedes aegypti mosquito
Source: BMC Genomics. 2016 Jan 6;17:32. doi: 10.1186/s12864-015-2239-0 (PMC4704297; doi:10.1186/s12864-015-2239-0)

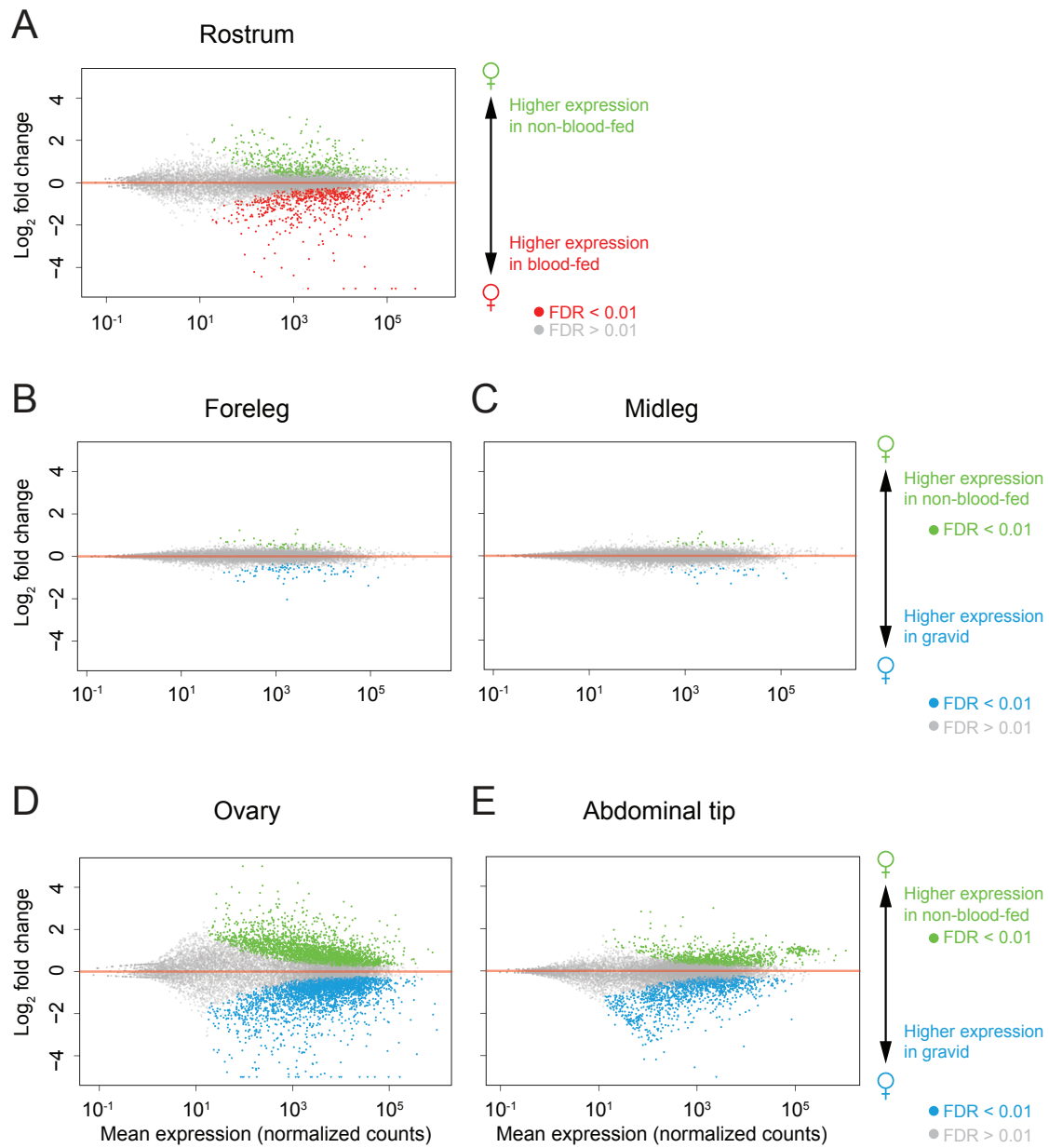

Supplement: Additional file 10: — Shows gene expression changes across the female gonotrophic cycle. MA plots of genes differentially expressed in non-blood-fed versus blood-fed female rostrum (a) or non-blood-fed versus gravid female forelegs (b), midlegs (c), ovary (d) and abdominal tip (e). Genes were identified as significantly regulated by a single-tissue comparison using DESeq2 (α < 0.01). (PDF 3876 kb) [file 12864_2015_2239_MOESM10_ESM.pdf]
